# Clinical validation of 3D-printed swabs in adults and children for SARS-CoV-2 detection

**DOI:** 10.1093/biomethods/bpad009

**Published:** 2023-06-07

**Authors:** Ana Laura Sanchez-Sandoval, Celia Sánchez-Pérez, José Antonio García-García, Silvia Plata Uriega-González, Guadalupe Mercedes Lucía Guerrero-Avendaño, Eira Valeria Barrón-Palma

**Affiliations:** Servicio de Medicina Genómica, Hospital General de México “Dr. Eduardo Liceaga”, Mexico City, Mexico; Instituto de Ciencias Aplicadas y Tecnología de la Universidad Nacional Autónoma de México, Mexico City, Mexico; Dirección de Educación y Capacitación en Salud. Hospital General de México “Dr. Eduardo Liceaga”, Mexico City, Mexico; Servicio de Pediatría, Hospital General de México “Dr. Eduardo Liceaga”, Mexico City, Mexico; Dirección General, Hospital General de México “Dr. Eduardo Liceaga”, Mexico City, Mexico; Servicio de Medicina Genómica, Hospital General de México “Dr. Eduardo Liceaga”, Mexico City, Mexico

**Keywords:** COVID-19, SARS-CoV-2, 3D-printed swabs, clinical validation, polylactic acid, thermoplastic elastomers

## Abstract

Throughout the entire coronavirus disease 19 (COVID-19) pandemic, there were disruptions in the supply chain of test materials around the world, primarily in poor- and middle-income countries. The use of 3D prints is an alternative to address swab supply shortages. In this study, the feasibility of the clinical use of 3D-printed swabs for oropharyngeal and nasopharyngeal sampling for the detection of SARS-CoV-2 infection was evaluated. For that purpose, paired samples with the 3D printed and the control swabs were taken from 42 adult patients and 10 pediatric patients, and the results obtained in the detection of SARS-CoV-2 by reverse transcription and quantitative polymerase chain reaction (RT-qPCR) were compared. Additionally, in those cases where the result was positive for SARS-CoV-2, the viral load was calculated by means of a mathematical algorithm proposed by us. For both adults and children, satisfactory results were obtained in the detection of SARS-CoV-2 by RT-qPCR; no significant differences were found in the quantification cycle values between the 3D-printed swab samples and the control samples. Furthermore, we corroborated that the 3D-printed swabs caused less discomfort and pain at the time of sampling. In conclusion, this study shows the feasibility of routinely using 3D-printed swabs for both adults and children. In this way, it is possible to maintain local and cheaper consumption along with fewer distribution difficulties.

## Introduction

On 31 December 2019, the World Health Organization (WHO) reported a series of pneumonia cases caused by an unknown agent in the city of Wuhan, Hubei province, China. In March 2020, it was granted the category of pandemic [1–3]. Since the publication of the first reported cases in December 2019, the disease produced by the SARS-CoV-2 virus (COVID-19) has caused, until April 2023 (about 1200 days after the start of the pandemic), more than 760 million cases and close to 6.9 million deaths globally [4].

The current COVID-19 pandemic poses health, social, and economic challenges of enormous magnitude. The accurate and efficient diagnosis of SARS-CoV-2 infection is critical, both from the epidemiological and clinical points of view [5, 6]. Diagnostic tests for COVID-19 are a cornerstone of pandemic control [7]. Identification, isolation, and contact tracing are critical strategies that have been used to control the spread of the disease.

Since the beginning of the pandemic, there have been supply chain interruptions that, coupled with the need for massive use of tests, have caused a great shortage of supplies to carry out diagnostic tests internationally, beginning with the shortage of sampling swabs, with the lack of nasopharyngeal (NS) swabs having a greater impact [8–10], becoming real bottlenecks in certain regions of the world [11–14]. One possible solution to this crisis is the development of 3D-printed NS and/or oropharyngeal (OP) swabs with quality and functionality equal to or even better than those from traditional swabs [9, 15].

To date, innovative 3D-printing technologies are used in the automotive, aerospace, clothing, pharmaceutical, and biomedical industries by creating pre-engineered and customized products. It has also been successfully used to efficiently address the disruption in the global medical equipment supply chain [16].

In this way, several materials have been used for the development of 3D-printed swab prototypes, including polyester and polylactic acid (PLA) [11], being the development of pediatric swabs a special challenge, as it must combine the effectiveness in sample collection, the safeness for children, and trying to abolish most of the inconvenience associated with the swabbing method [17, 18].

The aim of the present study was to perform a clinical validation of two prototypes of 3D-printed swabs, using PLA in OP and NS swabs for use in adults already probed in healthy patients [19] and thermoplastic elastomers (TPEs) for brand new pediatric swabs; comparing their performance against common rayon-tipped swabs routinely used in our hospital (Disposable Nasal/Throat Swab, BIOLOGIX^®^, China). It is important to point out that the same rayon-tipped swab model (control swab) has been used both for adults and children during the whole pandemic in public health hospitals. Also, following the recommendations of the national regulatory entities, the detection of the SARS-CoV-2 virus is carried out using combined NS and OP sampling placed into a single viral transport tube.

## Materials and methods

### Patient selection and sample collection

**Figure 1.**
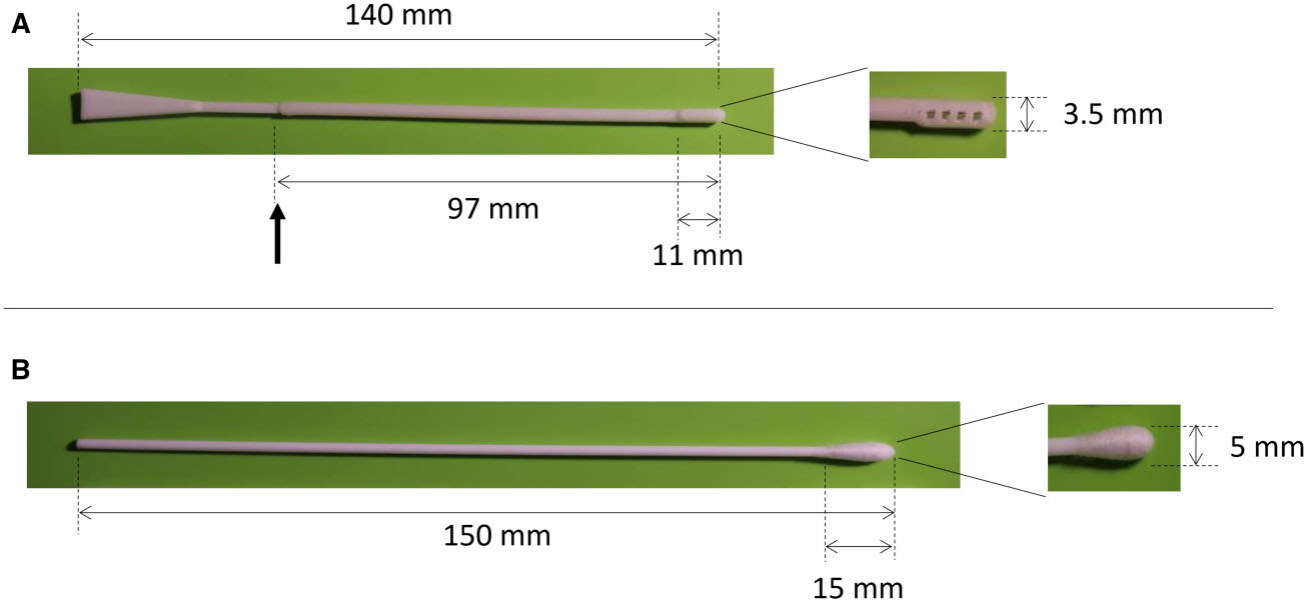
Pediatric 3D-printed and control swabs used in this study. (**A**) Pictures of the complete TPE 3D-printed swab and its collection head. The swab has a breakpoint (marked with thick arrow) to improve the handling. (**B**) Pictures of the complete control swab (with no breakpoint) and its collection head. Dimensions are described in the pictures.

The present study protocol was reviewed and approved by the Research, Ethics, and Biosafety Committees, of the General Hospital of Mexico “Dr. Eduardo Liceaga” (approval ID DI/20/501/04/52). The design and preclinical validation of PLA swabs for adults were previously reported. In brief, mechanical properties such as flexibility, breaking point, adaptability, and material stability, and biological properties such as sterility and skin reactivity were evaluated, as well as the ability to collect biological material in healthy individuals [19]. Following the same method that was used for the design and preclinical evaluation of the swab designed for adults, we decided to now produce and evaluate a swab made with TPE specifically designed for pediatric patients (Fig. 1).

Informed consent was submitted by all subjects when they were enrolled. In total, 52 paired samples were collected, both with the control swab and with the 3D-printed swabs (PLA or TPE, for adults or children, respectively), of OP and NS exudates. The samples were taken immediately one after the other. The study population consisted of symptomatic outpatients (*n* = 21), first-time symptomatic hospitalized patients (*n* = 22), hospitalized patients re-tested to analyze their clinical course (*n* = 4), and those for whom the SARS-CoV-2 test was requested as a prerequisite for a surgical procedure (*n* = 5). Within these samples, adult (*n* = 42) and pediatric (*n* = 10) patients were included. Considering the multiple challenges involved in sample collection in pediatric patients, it was determined that, for this group of patients, the NS and OP swabs were placed in an independent tube to perform the viral determination with each of the swabs separately. For all determinations, the viral transport medium was the same.

### Viral RNA isolation

Viral RNA was isolated from OP and NS samples using ExiPrep™ 96 Viral DNA/RNA kit (Bioneer, South Korea) and MagMAX™ Viral RNA isolation kit (Thermo Fisher Scientific, USA) according to the manufacturer’s specifications.

### SARS-CoV-2 detection

The SARS-CoV-2 detection was immediately performed after RNA isolation. Reverse transcription and quantitative polymerase chain reaction (RT-qPCR) were carried out using the GeneFinder™ COVID-19 Plus RealAmp kit (OSANG Healthcare, South Korea) according to the manufacturer’s protocol. The kit contains specific sets of primers designed to amplify three viral genes (*E*, *N*, and *RdRp*) of the SARS-CoV-2 genome, and a housekeeping human gene (IC: internal control) as a reference. SARS-CoV-2 positive results were given based on the amplification of the viral genes and the internal control gene with quantification cycle (Cq) values not higher than 40. Negative results were reported when only the *IC* gene was amplified with Cq values not higher than 35.

### Viral load determination

For the estimation of the relative viral load, we designed an algorithm making modifications to the original 2^−ΔΔCt^ qPCR analysis of relative gene expression [20]. The calculation was carried out in three steps. First, we calculated the delta Cq: where Cq_RdRp_ is the Cq value of the viral gene RdRp, and Cq_Ctrl_ is the Cq value of the IC gene. The purpose of the *IC* gene is to normalize the PCRs for the amount of RNA added to the reactions. Then, we calculated the viral gene expression relative to the *IC* gene (Rel V_ex_), thus eliminating the variation that may exist due to the sampling technique in each patient, and considering that the basic principle of a PCR reaction is that each reaction cycle doubles the copy number of the amplified gene, and therefore a difference of one cycle between the two Cq values indicates twice as many copies of one gene compared to the other:


$$\Delta \text{Cq}={\;\text{Cq}}_{\text{RdRp}}–{\text{Cq}}_{\text{Ctrl}}$$

$${\text{Rel}\;V}_{\text{ex}}={\;2}^{-\Delta \text{Cq}}$$

Finally, the relative viral load was calculated using a logarithmic scale to display the wide range of values in a compact way:


$$\text{Relative viral load}={\;\;\text{log}\;}_{10}\left({\text{Rel}\;V}_{\text{ex}}\right)$$

Once the numerical value of the viral load was obtained, cutoff points were established to define the viral load as low (values less than −1), medium (values between −1 and 1), and high (values >1). Such cutoffs were considered after analyzing the distribution of the relative viral load values obtained from more than one hundred SARS-CoV-2 positive RT-qPCR results from patients at this hospital (data not shown).

### Statistical analysis

General data were analyzed using descriptive statistics: mean, standard deviation, and the range of values for quantitative variables; percentages and frequencies for categorical variables. Inferential analysis was performed with mean differences for two related groups, both to assess the presence of local adverse events and to measure efficacy through the Cq values. The concordance between the positive/negative results in both samples (collected with the control swabs and with the 3D-printed swabs) and the variability in the Cq values between both samples were evaluated using the Kappa index. All the analysis was performed with SPSS statistical software V.26 (http://www-01.ibm.com/software/uk/analytics/spss/; RRID: SCR_002865), considering a statistical significance when *P *≤* *0.05.

## Results

A total of 52 patients participated in the study, 42 adults and 10 children. Table 1 shows some of the characteristics of the patients included in the study. Regarding hospitalization, the proportion of inpatients and outpatients was the same in the adult group. However, in the pediatric patient group, there was only one outpatient. This was due to the greater ease of recruiting hospitalized pediatric patients for this study, and not due to the clinical characteristics of the patients. Age and number of days between symptom onset and sample collection were obtained from clinical records.

**Table 1.** Characteristics of the adult and pediatric patients

Characteristic	Adult,^a^ *n* (%)	Pediatric,^b^ *n* (%)
Number of patients	42 (81)	10 (19)
Sex		
Female	20 (48)	6 (60)
Male	22 (52)	4 (40)
Type of patient		
Inpatients	20 (48)	9 (90)
Outpatients	22 (52)	1 (10)
Age^c^	45 (±18)	13 (±1)
Days between symptom onset and sample collection^c^	6 (±5)	2 (±1)
SARS-CoV-2 detection by RT-qPCR		
Positive	27 (64)	0 (0)
Negative	15 (36)	10 (100)

a Tested with PLA (PLA) 3D-printed swabs.

b Tested with TPE 3D-printed swabs.

c Mean ± standard deviation.

Percentages are rounded.

After taking the samples from the 52 patients, a questionnaire related to the discomfort caused by each type of swab used was carried out, obtaining the most favorable comments for the 3D-printed swab for NS samples. There were no accidents or incidents during the sampling with either of the two types of swabs.

Considering the handling of the samples in the RNA extraction process, a questionnaire was made to the laboratory staff, and according to the answers obtained, the 3D-printed swabs were evaluated as the most suitable for manipulating the sample, since they facilitate the handling of the viral transport medium tube at the time of taking the aliquot with a micropipette and reduce the risk of sample leakage.

In each of the cases, a positive or negative result for SARS-CoV-2 was obtained as described in the methodology. For all of the outpatients, first-time symptomatic hospitalized patients, and the pre-surgical patients, 100% concordance was found when comparing the results between the samples taken with the control swab and the ones taken with the 3D-printed swab. However, concerning the hospitalized patients re-tested to analyze their clinical course, two of the samples had different results: with the control swabs there was no amplification of the viral genes, whereas with the 3D-printed swab, the result was positive for SARS-CoV-2 (Table 2).

**Table 2.** Cq values of RT-qPCR assays of adult patients explored

Patient ID	Control swabs				3D-printed swabs			
	E	N	RdRp	IC	E	N	RdRp	IC
A-1	26.38	27.65	26.45	29.16	22.36	23.21	22.53	28.21
A-2	–	–	–	30.85	–	–	–	31.14
A-3	34.28	33.75	33.54	30.49		38.50	37.37	31.76
A-4	22.40	24.00	22.97	25.37	22.68	24.05	23.46	21.55
A-5	–	–	–	27.36	–	–	–	29.66
A-6	–	–	–	28.81	–	–	–	29.06
A-7	28.35	29.51	28.16	30.04	24.06	25.24	24.16	28.48
A-8	28.75	29.76	29.15	28.23	29.00	29.85	29.41	30.10
A-9	–	–	–	29.85	–	–	–	29.43
A-10	–	–	–	28.91	–	–	–	29.56
A-11	–	–	–	30.69	–	–	–	31.36
A-12	26.10	26.70	26.32	30.46	25.73	26.37	25.73	29.35
A-13	–	–	–	32.98	–	–	–	30.54
A-14	–	–	–	31.97	–	–	–	30.47
A-15	23.85	25.24	24.14	28.90	26.29	26.77	26.70	29.60
A-16	18.80	20.29	18.90	25.01	18.76	19.60	19.40	25.06
A-17	–	–	–	29.05	–	–	–	30.74
A-18	–	–	–	29.86	–	–	–	31.96
A-19	–	–	–	21.39	–	–	–	30.16
A-20	27.83	28.95	27.70	29.51	22.10	23.27	21.97	27.26
A-21	31.77	31.42	31.53	28.33	30.72	31.45	30.65	28.79
A-22	22.55	24.09	23.32	23.57	20.87	22.59	21.17	24.69
A-23	36.26	34.32	33.68	27.95	32.53	32.66	32.30	27.39
A-24	–	–	–	27.24	–	–	–	26.43
A-25	25.67	26.64	25.94	27.15	26.68	27.82	27.11	27.80
A-26	–	–	–	27.66	–	–	–	27.74
A-27	25.39	26.22	25.84	27.58	25.92	25.61	26.41	24.67
A-28	22.28	23.54	22.62	24.56	27.55	27.69	27.56	26.69
A-29	33.85	33.99	33.50	29.25	–	35.91	35.25	26.94
A-30	34.30	35.69	33.47	24.97	29.01	30.52	28.96	25.39
A-31	34.62	34.07	34.17	24.11	36.30	34.74	34.55	22.85
A-32	–	35.27	37.61	25.20		35.83	37.81	23.00
A-33	31.66	33.27	32.21	34.85	31.45	32.41	31.90	34.90
A-34	24.95	27.16	26.10	28.94	23.15	25.37	24.26	26.58
A-35	–	39.86	39.41	30.84	33.22	33.80	34.03	27.70
A-36	–	–	–	28.43	–	–	–	29.88
A-37	28.41	30.03	29.10	30.32	27.99	28.71	28.85	26.31
A-38	21.26	22.58	21.99	27.72	24.26	25.98	25.03	29.88
A-39^a^	–	–	–	27.55	31.72	31.87	31.21	25.02
A-40^a^	–	–	–	25.96	–	38.35	35.92	30.47
A-41	–	–	–	29.82	–	–	–	29.40
A-42	–	35.51	35.13	25.82	–	–	37.40	29.21

a Cases in which the RT-qPCR test was negative in the samples obtained with the control swabs, but positive in the samples obtained with the 3D-printed swabs.

**Figure 2.**
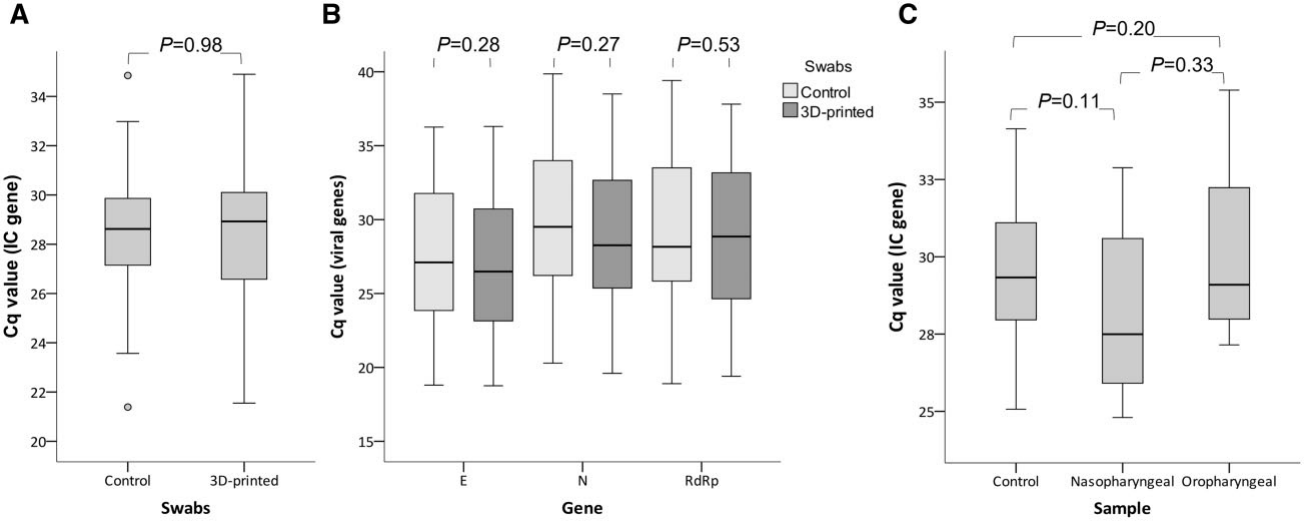
Comparative box plots between Cq values of RT-qPCR experiments. (**A**) IC gene (internal control) Cq values of samples taken with the control swabs and the 3D-printed swabs. (**B**) Cq values of the viral genes (*E*, *N*, and *RdRp*) of samples taken with the control swabs and the 3D-printed swabs. (**C**) IC gene Cq values of pediatric samples taken with the control swabs (NS and OP together), the 3D-printed NS swab, and the 3D-printed OP swab. *P*-values are shown for each comparison. The middle line of each box represents the median of the Cq values.

For the comparative analysis between the Cq values of the samples taken with control swabs and 3D-printed swabs, the comparison was first made only between the Cq values of the adult patient samples (Table 2), since in this case, each sample consisted of a combined NS and OP sampling. When comparing the abundance of the *IC* gene, no significant difference was found (*P *=* *0.98; Fig. 2A). Then, the Cq values of the viral genes (*E*, *N*, and *RdRp*) of the positive samples were compared, finding no significant differences for any gene (*P *=* *0.28, *P *=* *0.27, and *P *=* *0.53, respectively, Fig. 2B).

On the other hand, the analysis of the pediatric samples (Table 3) was done by comparing the Cq values of the *IC* gene of three different types of sampling: the conventional sample (NS and OP swabs in the same tube of viral transport medium using the control swabs); a sample with only the NS exudate taken with a 3D-printed swab; and another with only the OP exudate taken with a 3D-printed swab, finding no significant differences between the Cq values of the three different samples (*P *=* *0.64, ANOVA test), even considering that only one swab was used in the 3D-printed swab samplings. When comparing the Cq values of the NS and OP exudates, both with the 3D-printed swabs, we did not find significant differences either (*P *=* *0.33, Fig. 2C); however, the Cq values of the OP swabs were mostly higher than the NS, suggesting a greater amount of biological material taken with the latter (Table 3). It is worth mentioning that, in the case of pediatric patients, only the Cq value of the *IC* gene was compared between samples, since none of them were positive for SARS-CoV-2.

**Table 3.** Cq values of RT-PCR assays of pediatric patients explored

Patient ID	Cq value of IC gene		
	Control swabs	3D-printed NS swabs	3D-printed OP swabs
P-1	34.14	32.88	35.39
P-2	33.60	30.59	34.65
P-3	26.67	27.25	27.15
P-4	29.31	31.92	29.48
P-5	31.10	24.80	29.82
P-6	28.66	25.91	34.07
P-7	27.96	28.23	28.43
P-8	25.07	25.65	27.54
P-9	29.35	26.10	28.71
P-10	29.54	27.74	37.86

**Figure 3.**
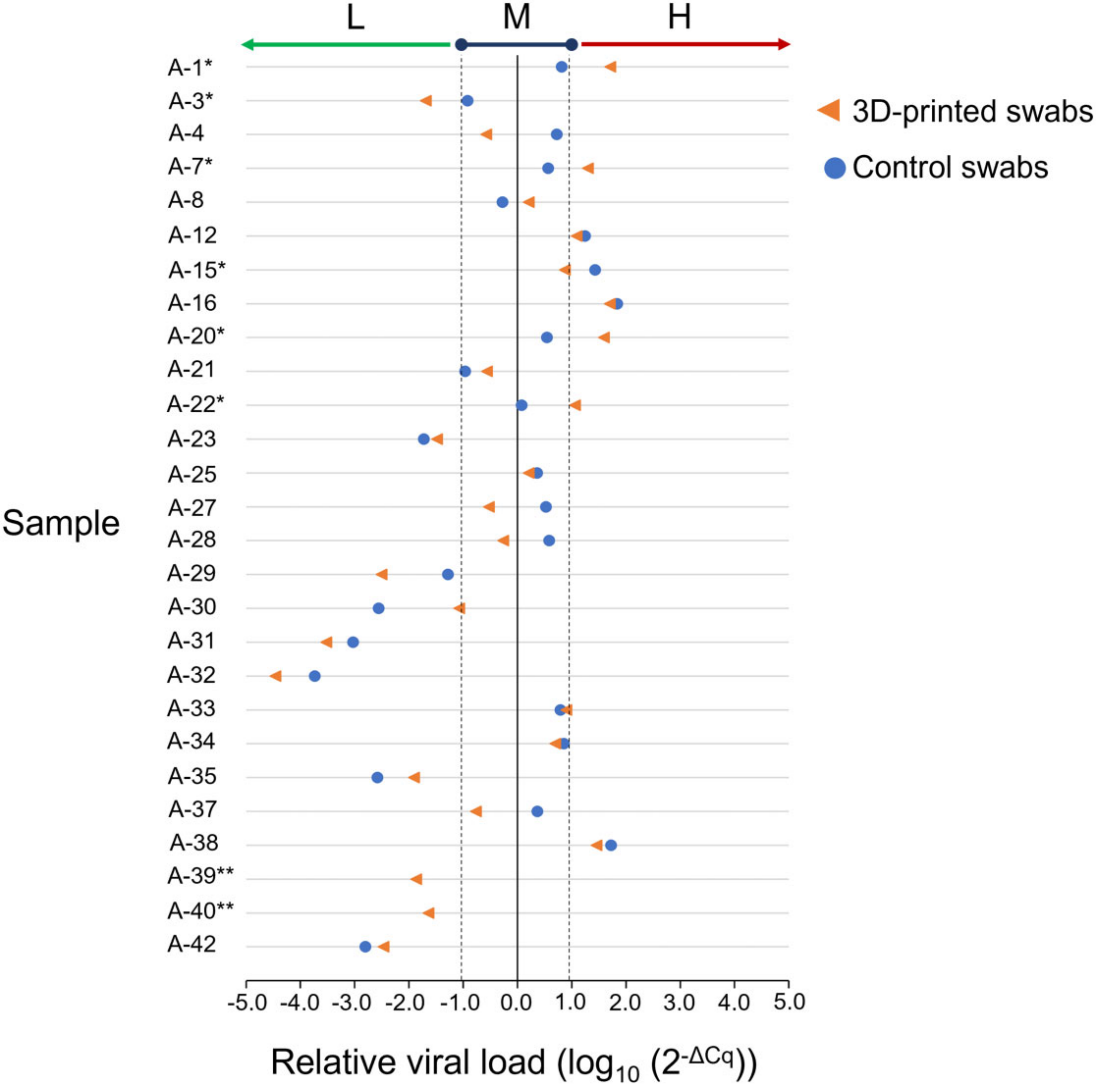
Relative viral load in positive samples detected with 3D-printed and control swabs. The relative viral load of the samples obtained for each patient with the 3D-printed swab (orange triangles) and the control swab (blue circles) are represented in each horizontal line. Values < −1 = low viral load (L), values between −1 and 1 = medium viral load (M), values > 1 = high viral load (H). Vertical dotted lines indicate the cut-offs.

Regarding the analysis of the viral load, the quantification of 27 positive samples that were taken with the 3D-printed swabs and 25 positive samples taken with the control swabs was carried out (Table 4), classifying their viral load as low, medium, or high. When comparing the results obtained between the samples taken with the control swabs and those taken with the 3D-printed swabs, the coincidence was 70.4% (19 samples). In six samples (22.2%), there was no coincidence in the viral load classification (Fig. 3, samples marked with an asterisk); four of these cases were classified as having a lower viral load when the sample was taken with the control swab, compared with the viral load classification obtained with a 3D-printed swab (samples A-1, A-7, A-20, and A-22).

**Table 4.** Viral load calculated with log_10_ (2^-ΔCq^) algorithm

Sample ID	Relative viral load	
	Control swab	3D-printed swab
A-1	0.82	1.71
A-3	−0.92	−1.69
A-4	0.72	−0.57
A-7	0.57	1.30
A-8	−0.28	0.21
A-12	1.25	1.09
A-15	1.43	0.87
A-16	1.84	1.70
A-20	0.54	1.59
A-21	−0.96	−0.56
A-22	0.08	1.06
A-23	−1.72	−1.48
A-25	0.36	0.21
A-27	0.52	−0.52
A-28	0.58	−0.26
A-29	−1.28	−2.50
A-30	−2.56	−1.07
A-31	−3.03	−3.52
A-32	−3.74	−4.46
A-33	0.79	0.90
A-34	0.85	0.70
A-35	−2.58	−1.91
A-37	0.37	−0.76
A-38	1.72	1.46
A-39^a^	ND	−1.86
A-40^a^	ND	−1.64
A-42	−2.80	−2.47

a Cases in which the RT-qPCR test was negative in the samples taken with the control swabs, but positive when taken with the 3D-printed swabs.

ND = not determined. The sample was negative.

On the other hand, in the two cases where the viral genes were detected using the 3D-printed swabs but not with the control swabs, it can be seen that the calculated viral load was low (Table 4, Fig. 3, samples marked with two asterisks), although the miss-correlation cannot be attributed to this fact, as in some of the other samples with even lower viral loads, the results obtained with both swabs do correlate (e.g. A-31, A-32, A-35, A-42).

The performance results of the PLA adult swabs against the control swabs were as follows: sensitivity 100%, specificity 100%, positive predictive value 100%, negative predictive value 100%, and accuracy 100%. It is important to note that the performance of the PLA swab was better since the sensitivity of the control swab was 92%, the negative predictive value was 87%, and the accuracy was 95%.

The performance results of the TPE pediatric swabs against the control swabs were as follows: specificity 100%, negative predictive value 100%, and accuracy 100%. Since there were no positive cases among the group of pediatric patients, it was not possible to calculate the sensitivity and the positive predictive value.

## Discussion

The COVID-19 pandemic caused a crisis in health systems around the world. One of the critical points was the decrease (sometimes the absence) of supplies to meet the protection needs of frontline healthcare workers, as well as the need of improving the diagnosis and treatment of patients. To face this crisis, an unprecedented collaborative trans-disciplinary effort was developed in different regions of the world, including the use of 3D-printing technologies [21].

To date, ground-breaking 3D-printing technologies are used in automobile, aerospace, clothing, pharmacological, and biomedical industries to cite a few examples. The use of 3D printing in medicine is not new, with consistent and increasing applications in different fields of healthcare [22]. The main reason for using these technologies is due to the popularity of 3D printers, the low cost of printing, and its versatility [23]. Some examples of 3D printing used in the COVID-19 response are N95 respirator masks, face shields, swabs, ventilator valves, and drug manufacturing [24].

The molecular identification process of any infectious agent, in this case SARS-CoV-2, is successful thanks to the fact that all stages are considered, including the sampling, genetic material extraction, RT-qPCR reaction, and analysis. In the sample collection process, it is desirable to use swabs that generate the least amount of discomfort for the patient, and that are also comfortable for the qualified personnel who take the sample and for the people who process it for RNA extraction. Adequate swabs also avoid risks of contamination (samples and personnel) when handling.

The 3D-printed swabs emerged as a plausible solution to their shortage during the current pandemic, since they have proven to be a reliable, cost-effective, and fast solution for COVID-19 testing and with the capacity to mitigate substantial supply chain obstacles with growing testing requirements [25, 26].

Currently, different printing technologies have been tested for the manufacture of 3D-printed swabs; the ones mainly used are stereolithography, Selective Laser Sintering, Digital Light Processing, and Fused Deposition Modeling (FDM). The modeling of the 3D prints varies according to the type of printer and software used. In this study, the modeling of the swabs was carried out considering not only the ability of the swab to collect a large number of cells but also the anatomical characteristics of adult and pediatric patients. Our swabs were printed using the FDM system, which has the advantage of being cheaper compared to other printing systems; in addition, the low-cost polymers used in the manufacture of our swabs have been shown to be biodegradable, non-toxic, and validated through mechanical and biological tests, without interfering with the PCR reaction [19, 25, 26].

There are several protocols for taking samples in adult and pediatric patients; however, in institutions such as ours, the protocol indicates that the sample must be taken with a double swab (NP and OP) per patient in order to obtain more biological material for reliable detection of the virus. In adult patients, this condition was maintained, but the effectiveness and comfort of using the 3D-printed swabs were demonstrated.

As for pediatric patients, a single sample taken with a suitable swab is sufficient to conduct viral detection, thus avoiding further discomfort in patients. Even though the small number of pediatric patients was one of the limitations of the study, this trial demonstrates that a reliable result can be obtained from a single NS swab taken with the 3D-printed swab in these patients.

In this study, the results indicate that, with the 3D-printed swabs, sufficient and good quality biological material is obtained, allowing nucleic acid amplification by RT-qPCR. In the case of the two patients with a negative result in the RT-qPCR test with the control swabs and who were positive in the collection of material with the 3D-printed swabs, this difference could indicate that, because of its greater flexibility and thinness, the 3D-printed prototype swab is more efficient, facilitating the sample collection mainly for the NS exudate by reaching deeper areas of the oropharynx. It is important to highlight that these two patients had a positive diagnosis of SARS-CoV-2 by RT-qPCR performed 11–12 days before conducting our study, suggesting that at the time of doing the tests with the 3D-printed swabs, the patients were already recovering from the disease, showing the importance of taking the sample from the correct tissues (in this case the oropharynx) to reach an accurate diagnosis. This is not easy to achieve with the control swabs, due to their size and low flexibility, but it can be easily achieved with the 3D-printed swabs.

In the present study, we analyzed the effectiveness and sensitivity of swabs through RT-qPCR tests for SARS-CoV-2, due to the context in which we found ourselves in our hospital at the time. However, our results indicate that these 3D-printed swabs can be used not only for samples that are going to be subjected to a diagnosis by means of RT-qPCR but also for other molecular tests such as reverse transcription loop-mediated isothermal amplification, antigen tests, and sequencing, to name a few, some of which can even be cheaper and faster than the RT-qPCR test, with the possibility of further benefit to the most economically vulnerable populations.

In conclusion, the use of PLA (for adults) and TPE (for pediatrics) 3D-printed swabs in sample collection for SARS-CoV-2 testing or other genetic markers, either from microorganisms or from the patient itself, is feasible for both adults and children and convenient given the shortage of supplies. This design can be easily adopted in countries where commercial swabs are not readily available and can play a vital role in public health efforts for disease control, primarily in low-income countries, not only for the current pandemic but also for the ones to come, as well as for other routinary tests where OP and/or NS samples are needed.

## Data Availability

The data underlying this article will be shared on reasonable request to the corresponding author.
